# Impact of surgical technique on hemodynamic instability in patients with pheochromocytoma: a single-centre retrospective cohort study

**DOI:** 10.1007/s00464-025-11794-2

**Published:** 2025-05-15

**Authors:** Amir-Hossein Chaman Baz, Julie van de Wal, Simone A. A. Willems, Frank d’Ancona, Xiaoye Zhu, Henri J. L. M. Timmers, Johan F. Langenhuijsen

**Affiliations:** 1 Department of Urology, Radboud University Medical Centre, Geert Grooteplein Zuid 10, 6525 GA Nijmegen, The Netherlands; 2 Department of Anaesthesiology, Radboud University Medical Centre, Nijmegen, The Netherlands; 3 Department of Internal Medicine, Section Endocrinology, Radboud University Medical Centre, Nijmegen, The Netherlands

**Keywords:** Endoscopic surgery, Pheochromocytoma, Hemodynamic instability, Transperitoneal laparoscopic adrenalectomy, Posterior retroperitoneoscopic adrenalectomy, Cohort study

## Abstract

**Background:**

Endoscopic adrenalectomy by either transperitoneal laparoscopic (TLA) or posterior retroperitoneoscopic approach (PRA) is the preferred treatment for pheochromocytoma (PCC). PRA shows advantages in patient outcome, but blood pressure fluctuations may occur due to limited working space and increased CO_2_-pressure. We investigated the impact of surgical technique on intraoperative hemodynamic instability in patients with PCC.

**Methods:**

Patients who had endoscopic adrenalectomy for PCC consecutively from 2007 to 2022 were included in this retrospective cohort study. The primary outcome was hemodynamic instability (HI-score) and secondary outcomes were hemodynamic parameters and drug administration.

**Results:**

Overall, 101 patients met the inclusion criteria, 57 had TLA and 44 PRA. The two groups were similar in baseline characteristics. The HI-score was higher in PRA than in TLA (97 vs 46, *p* < 0.001) due to more frequent (IQR: 2–5 vs IQR: 1–3, *p *= 0.025) and longer episodes of hypotension (5.6% vs 7.1%, *p* = 0.013), and longer episodes of bradycardia (9.9% vs 16.9%, *p* = 0.038). On the contrary, TLA patients had higher maximum systolic blood pressure (169 mmHg vs 157 mmHg, *p* = 0.046), more frequent episodes of tachycardia (31.6% vs 6.8%, *p* = 0.002) and higher maximum heart rate (90 bpm vs 80 bpm, *p* = 0.024). PRA patients needed more vasoconstrictive drugs (97.7% vs 78.9%, *p* = 0.017) and fluid infusion (1111 ml/h vs 798 ml/h, *p* = 0.004), whereas TLA patients received more vasodilating drugs (64.9% vs 38.6%, *p* = 0.009).

**Conclusions:**

PRA was associated with higher hemodynamic instability than TLA reflected by hypotension, need for vasoconstrictive drugs and fluid infusion in a selected cohort of patients with pheochromocytoma.

**Graphical abstract:**

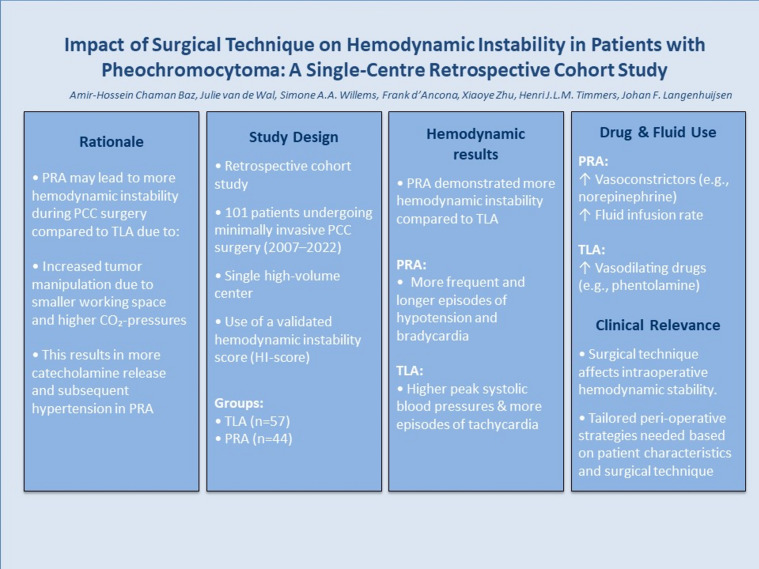

**Supplementary Information:**

The online version contains supplementary material available at 10.1007/s00464-025-11794-2.

Pheochromocytomas (PCCs) are rare catecholamine producing tumours with an incidence of 0.6 per 100,000 cases [1, 2]. These tumours cause a range of symptoms, including headaches, palpitations, and excessive sweating, due to the episodic release of excessive amounts of catecholamines [3]. Furthermore, these tumours can lead to life-threatening complications, such as hypertensive crisis, arrhythmias, cerebrovascular events and myocardial infarction [4, 5].

The corner stone of therapy for PCC is surgical resection. Endoscopic surgical techniques, including transperitoneal laparoscopic adrenalectomy (TLA) and posterior retroperitoneoscopic adrenalectomy (PRA), are commonly used, but occasionally open surgery is still necessary. Adequate preoperative preparation, including alpha-adrenergic blockade to control blood pressure and prevent intraoperative hypertensive crisis caused by catecholamine release which is triggered by intubation and tumour manipulation, is advocated in international guidelines [6–8].

TLA is most commonly used due to its ability to provide ample working space during PCC resection, with clear anatomical landmarks, and adequate manoeuvrability of surgical instruments. However, it has a potential risk of complications involving abdominal organs and longer operating times compared to PRA [9]. PRA allows for direct access and dissection avoiding the abdominal organs and the implications of pneumoperitoneum, such as cardiopulmonary and CO_2_-absorption effects. In previous studies PRA is suggested to result in shorter operating times and hospital stay, as well as lower blood loss, and less postoperative pain compared to the TLA. However, these advantages may vary depending on surgical expertise and patient characteristics [9]. Additionally, PRA is less widespread because of its technical challenges with limited retroperitoneal working space and the lack of anatomical landmarks [10]. Theoretically, this may increase tumour manipulation during resection, subsequent catecholamine release and ensuing increase in blood pressure and heart rate (HR). Additionally, higher CO_2_-pressure levels are frequently used in PRA compared to the TLA (20 vs 12 mmHg) potentially causing mechanical stimulation of the tumour. Furthermore, the adrenal vein, which is situated anteriorly, is ligated at a later stage as compared to TLA. We hypothesize that these differences between TLA and PRA will differentially affect intraoperative blood pressure and HR fluctuations. This may have potential clinical consequences, because hemodynamic instability, is a major concern in PCC surgery as it is associated with an increased risk of postoperative complications, due to fluctuations in blood pressure affecting organ perfusion and recovery [11]. The aim of this study is to investigate the influence of these surgical techniques on intraoperative hemodynamic instability in PCC patients. Improved understanding of hemodynamic differences between surgical techniques may help optimize perioperative management, thereby reducing the incidence of adverse outcomes and associated healthcare costs.

## Materials and methods

This is a single-centre retrospective cohort study. The STROBE statement (Strengthening the Reporting of Observational Studies in Epidemiology) was utilized to report our findings in a comprehensive and transparent manner and was in line with the STROCCS guidelines [12, 13]. There was no predefined study protocol established. This study was approved by Medical Ethics Committee East-Netherlands (METC Oost-Nederland; reference number 2021–8291) and the need for formal informed consent was waived.

### Patient selection

Consecutive patients who underwent endoscopic adrenalectomy for PCC were included from a prospectively collected database from 2007 to 2022. All patients were over 18 years of age and operated by TLA or PRA technique. Patients with missing intraoperative data due to incomplete pre-electronic patient files (before 2014), after conversion to open adrenalectomy or switch to another endoscopic technique, or patients for partial or bilateral adrenalectomy and combined surgical procedures, were excluded to ensure a reliable comparison between surgical groups.

### Preoperative pharmacological management

The perioperative care is based on The Endocrine Society guidelines for PCC and paraganglioma [14]. All patients with a suspicion of PCC underwent biochemical screening, in case of positive biochemical screening, imaging with preferably CT-scan was performed to locate the tumour. In general, surgical resection was planned within 8 weeks after diagnosing PCC. Patients were preoperatively treated for 14 days with alpha-adrenoreceptor blockers, either phenoxybenzamine or doxazosin, and additional beta-adrenergic blockade and calcium channel antagonist when needed, according to a standardized approach as described previously [15]. Following the inclusion of the so-called PRESCRIPT trial, which demonstrates the non-inferiority of doxazosin for blood pressure control (MAP > 60 mmHg and SBP < 160 mmHg) compared with phenoxybenzamine, the choice of alpha-adrenergic blockade was changed from phenoxybenzamine to routine use of doxazosin from 2013 onwards due to costs [15].

### Operative techniques

All adrenalectomies were performed in Radboudumc, a high-volume center for adrenal surgery. Surgery was performed by three urologists with extensive experience in endoscopic surgery and adrenalectomy, two of whom were proficient in both techniques, while one specialized in TLA only, see Supplementary Table 1. Before 2011 patients routinely underwent TLA in our center for PCC. Thereafter, PRA was the preferred surgical technique for tumours ≤ 7 cm in patients with BMI of < 35 kg/m^2^. However, there was no prespecified protocol for selecting TLA or PRA, and the final decision remained at the surgeon’s discretion. A strict anaesthetic protocol is followed preoperatively and intraoperatively for each patient; refer to the supplementary data for detailed information.

### Posterior retroperitoneoscopic adrenalectomy

During PRA, patients were placed in the jack-knife position under general anaesthesia. Three trocars were placed: a 10 mm balloon trocar at the tip of the 12th rib, a 5 mm trocar at the tip of the 11th rib and an 11 mm trocar just below the costophrenic triangle. The pneumoretroperitoneum was established at an insufflation pressure of 20 mmHg CO2, followed by the development of the retroperitoneal space. Gerota’s fascia was then opened, and the upper pole of the kidney mobilised. Next, the planes of the adrenal gland were dissected with Harmonic™ 1100 Shears (Ethicon, Raritan, New Jersey, U.S.) and finally the adrenal vein was identified, clipped, and transected. The adrenal gland was extracted and the fascia and skin were closed.

### Transperitoneal laparoscopic adrenalectomy

During TLA, patients were placed in the lateral decubitus position under general anaesthesia. A Hasson trocar was introduced by open technique craniolaterally from the umbilicus and pneumoperitoneum was established at an insufflation pressure of 12 mmHg CO2, after which, under visual guidance, a 5 mm and 10–12 mm trocar were placed in the abdomen, and an additional 5 mm trocar above the anterior iliac crest on demand. Liver mobilisation was performed for right sided and spleen and pancreas mobilisation for left sided adrenalectomy. The adrenal gland was localised and preferably the adrenal vein was identified first, clipped and transected. Harmonic™ 1100 Shears (Ethicon, Raritan, New Jersey, U.S.) were used to dissect the planes between the adrenal gland and the kidney. The adrenal gland was extracted and the fascia and skin were closed.

### Postoperative care

During the postoperative phase, efforts were made to achieve extubation and removal of the gastric tube in the operating room. Afterwards, the patient was monitored for a duration of maximally 24 h in the Post-Anaesthesia Care Unit (PACU) or intensive care unit.

### Data collection and analysis of hemodynamic parameters

Patient data, which were obtained from EPIC (Epic Systems, Verona, WI, US), included preoperative work-up and blood pressure measurements. Prior cardiovascular events included: coronary artery disease, arrhythmias, heart failure, stroke, peripheral artery disease, aortic aneurysm, left ventricular hypertrophy, and transient ischemic attack. Additionally, continuous intraoperative hemodynamic data, including details on drug administration, blood pressure and heard rate measurements, were collected from anaesthesia reports. A validated scoring system, the HI-score, was used to assess hemodynamic instability using multiple variables including intraoperative hemodynamic parameters, use of vasoactive medication and fluid management [16]. An automated HI-score calculator was integrated into the database, facilitating the calculation and reporting of the HI-score upon collection of the necessary variables.

We applied defined criteria for hypertension, hypotension, tachycardia, and bradycardia in accordance with the specific thresholds outlined in the anaesthesiologic protocol seen above and used in the PRESCRIPT trial [16]. Postoperative complications were assessed within 30 days following surgery and were classified according to the Clavien-Dindo classification system.

### Statistical analysis

A Shapiro–Wilk test was performed on all parameters to test for normality. If the data had a normal distribution, they were reported with means and the 95%-confidence interval (CI-95%). In case of not normally distributed data, medians and the interquartile range (IQR) were reported. A T-test or Mann–Whitney-U test was performed to compare means or medians of both groups. Chi-squared test was performed to compare percentages in groups. We calculated a Spearman’s correlation coefficient to identify any correlations. We conducted subgroup analyses to assess the impact of alpha-blockade and PRA learning curve on the HI-scores.

Statistical analyses were performed with the IBM SPSS Statistics 26.0 software (IBM Corp, Armonk, NY, US). Figures and graphs were generated with Microsoft Excel (Microsoft, Redmond, Washington, US).

## Results

A total of 159 patients underwent endoscopic adrenalectomy for PCC, of whom 101 patients (63.5%) met the inclusion criteria. Reasons for exclusion were incomplete medical records in 32 patients, conversion to open surgery in 4 patients, intraoperative change to another endoscopic technique (from PRA to TLA) due to limited surgical space in 13 patients, and bilateral adrenalectomy or combined surgical procedures in 9 patients.

### Patient characteristics

A total of 57 patients had TLA and 44 patients PRA. The baseline characteristics of the patients in each group are shown in Table 1. The two groups were similar in terms of baseline clinical and tumour characteristics. Notably, the TLA group had a higher rate of preoperative cardiovascular events. Preoperative hemodynamic variables were similar between the groups, although the TLA patients received phenoxybenzamine more frequently than doxazosin.

**Table 1:** Group characteristics

		Surgical technique		
	Total	TLA *n* = *57*	PRA *n *= 44	p-value
Age (years), mean ± SD	55 (43.5–68)	56 ± 15	51 ± 14	0.097
Female, n (%)	56 (55%)	32 (56%)	24 (55%)	0.873
BMI (Kg/m^2^), median ± IQR	25.6 (22.9–28.9)	26.0 (22.8–29.6)	25.0 (23.1–28.1)	0.236
Smoking				0.553
Current, n (%)	18 (17.8%)	12 (21.1%)	6 (13.6%)	
Previous, n (%)	33 (32.7%)	19 (33.3%)	14 (31.8%)	
Never, n (%)	50 (49.5%)	26 (45.6%)	24 (54.5%)	
Prior cardiovascular event, n (%)	25 (24.8%)	19 (33.3%)	6 (13.6%)	0.023
ASA				0.702
I, n (%)	7 (6.9%)	4 (7.0%)	3 (6.8%)	
II, n (%)	47 (46.5%)	26 (45.6%)	21 (47.7%)	
III, n (%)	46 (45.5%)	27 (47.4%)	19 (43.2%)	
IV, n (%)	1 (1%)		1 (2.3%)	
Germline mutation				0.608
Yes, n (%)	13 (12.9%)	9 (15.8%)	4 (9.1%)	
No, n (%)	66 (65.3%)	36 (63.2%)	30 (68.2%)	
Not assessed. n (%)	22 (21.8%)	12 (21.1%)	10 (22.7%)	
Tumor localization, left, n (%)	53 (52.5%)	28 (49%)	25 (56.8%)	0.443
Maximum tumor diameter (mm), median (IQR)	37 (29–51)	40 (30–57)	36 (26–45)	0.214
Biochemical profile				
Total metanephrines (pmol/l), median (IQR)	5171 (2305–11257)	4850 (2245–9337)	5673 (2490–12005)	0.338
Pretreatment medication on day before surgery				
Phenoxybenzamine				
Number, n, (%)	23 (22.8%)	19 (33.3%)	4 (9.1%)	0.004
Total dose,mg, median (IQR)	100 (80–120)	100 (90–120)	85 (62.5–115.0)	0.456
Doxazosin				
Number, n, (%) Total dosemg, median (IQR)	78 (77.2%) 45 (32–48)	38 (66.7%) 40 (28–48)	40 (90.9%) 47 (33–48)	0.004 0.685
Use of CCA, n (%)	35 (34.7%)	19 (33.3%)	16 (36.4%)	0.751
Amlodipine				
Number n, (%)	16 (15.8%)	7 (12.3%)	9 (22.5%)	0.265
Total dose mg, median (IQR)	10 (6.25–10)	10 (10)	10 (5–10)	0.758
Nifedipine				
Number, n, (%)	19 (18.8%)	12 (21.1%)	7 (15.9%)	0.512
Total dose mg, median (IQR)	30 (30–60)	50 (30–60)	30 (30–60)	0.592
Use of beta-blockers, n (%)	93 (92.1%)	54 (94.7%)	39 (88.6%)	0.260
Metoprolol				
Number n, (%)	90 (89.1%)	51 (54.8%)	39 (88,6%)	0.893
Total dose mg, median (IQR)	87.5 (50–100)	100 (50–150)	75 (50–100)	0.539
Atenolol				
Number n, (%)	3 (3.0%)	3 (3.2%)	0 (0%)	0.122
Total dose (mg), median (IQR)	50 (25–50)	50 (25–50)	0	
Hemodynamic variables on day before surgery				
Supine				
SBP (mmHg), median (IQR)	124 (114–134)	124 (114–134)	125 (114–137)	0.506
DBP (mmHg), median (IQR)	73 (63–81.5)	73 (63–81.5)	73 (66–82)	0.506
HR (bpm), median (IQR)	75 (67–85.5)	75 (67–85.5)	75 (69–87)	0.445
Upright				
SBP (mmHg), median (IQR)	121 (110–133)	121 (110–133)	124 (104–142)	0.992
DBP (mmHg), median (IQR)	70 (61.5–80.5)	70 (61.5–80.5)	72 (64–80)	0.691
HR (bpm), median (IQR)	86 (76–95.5)	86 (76–95.5)	86 (74–96)	0.810

*BMI* Body mass index, *IQR* interquartile range

### Surgical outcomes

Table 2 presents the surgical outcomes of PRA and TLA. Operating times in PRA were significantly shorter with less blood loss. The postoperative hospital stay and complication rates were similar in both groups. Complications occurred in 10 patients, 4 of which were cardiovascular in nature and occurred in the TLA group, see Table 3.

**Table 2:** Comparison of surgical outcomes between TLA and PRA

	Surgical technique		
	TLA *n* = *57*	PRA *n* = *44*	*p-*value
Anesthesia duration (min), median (IQR)	159 (138.5–185)	132 (112–152)	< 0.001
Surgical duration (min), mean (95%-CI)	117 (108–124)	85 (86.6–91.4)	< 0.001
Blood loss (ml), median (IQR)	50 (15–135)	10 (5–20)	< 0.001
Postoperative hospital admission (days), mean (95%-CI)	4.4 (3.9–4.82)	3.8 (3.5–4.1)	0.053
Number of patients with longer ICU stay, n (%)*	3	1	0.445
Postoperative Complications, n (%)	7 (6.9%)	3 (3.0%)	0.362
Clavien-Dindo scale			0.18
1–2, n	4	3	
3, n	2		
4, n	1		

*TLA* Transperitoneal laparoscopic adrenalectomy, *PRA* posterior retroperitoneoscopic adrenalectomy, *IQR* interquartile range, *min* minute, *ICU* intensive care unit

*: If patients are admitted to the ICU postoperative for more than one day (per-protocol)

**Table 3:** Postoperative complications with Clavien-Dindo scale and specifications

Surgical technique	Clavien-Dindo scale	Complication
TLA	2	Hypertensive crisis (SBP > 180 mmHg), normalized after initiation of antihypertensive medication
TLA	1	Atrial fibrillation, managed conservatively without the need for medication or intervention
TLA	2	Persisting tachycardia (105–110 bpm), necessitating treatment with β-blockade
TLA	4b	Total small bowel ischemia 14 days postoperatively, prompting a laparotomy. Upon surgical intervention, necrosis was identified in the small intestine, extending from the jejunum to 40 cm proximal to the ileocecal junction and no pulsations were detectable in the superior mesenteric artery and celiac trunk. Consequently, a complete resection of the small bowel was undertaken. A retrospective analysis of the preoperative contrast-enhanced CT-scan of the abdomen, which was made in the work-up of adrenal disease, showed signs of arterial stenosis in both the SMA and celiac trunk
PRA	2	Pneumonia necessitating the administration of antibiotic therapy
TLA	3b	Suspected small bowel perforation requiring surgical intervention
TLA	3b	Incisional hernia, requiring surgical repair
TLA	2	Pneumonia, requiring antibiotic therapy
PRA	2	Suspected gallbladder stone, pain diminished after readmission and pain medication
PRA	2	The patient experienced abdominal pain necessitating the use of pain medication. The cause of the pain could not be identified

*TLA* Transperitoneal laparoscopic adrenalectomy, *PRA* posterior retroperitoneoscopic adrenalectomy

**Fig. 1:**
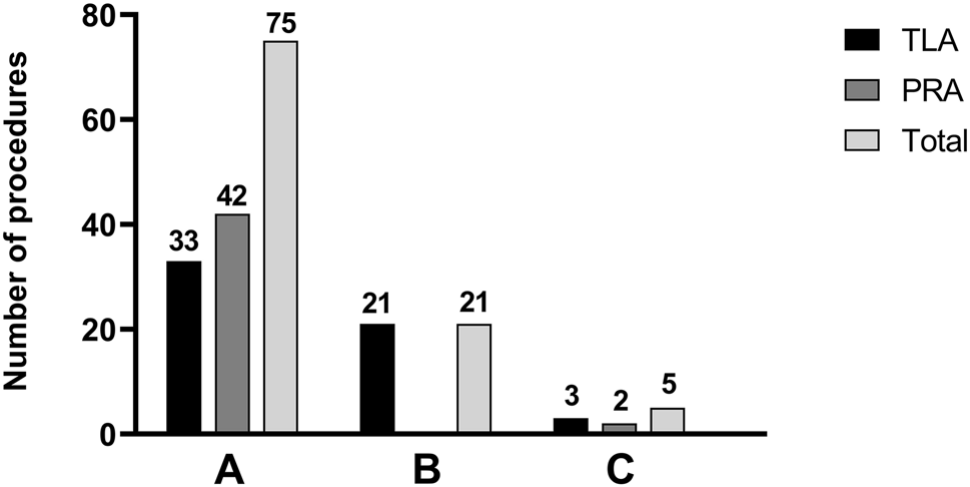
Number of TLA and PRA performed per urologist

Surgery was performed by three urologists, each with extensive experience in endoscopic surgery and adrenalectomy. Two urologists (A and C) were proficient in both techniques, while one (B) performed TLA only, see Fig. 1. The reasons for choosing TLA are shown in Table 4. In three cases, specific patient-related reasons led to the choice for TLA to achieve optimal exposure, including: partial adrenalectomy, lymph node involvement, and suspicion of ACC in a biochemically silent PCC.

**Table 4:** Reasons for the choice of TLA

Reasons	Frequency n, (%)	
Surgeon’s expertise		16 (28%)
Tumour size > 7 cm		12 (21%)
BMI > 35 kg/m^2^		10 (18%)
PRA was not introduced		9 (16%)
Space restrictions preoperatively		8 (14%)
Other reasons		3 (8%)

### Hemodynamic outcomes

In Table 5 the hemodynamic outcomes are presented. The PRA group had a significantly higher HI-score compared to the TLA group.

**Table 5:** Comparison of hemodynamic outcomes between the surgical techniques

	Surgical technique		
	TLA *n* = *57*	PRA *n* = *44*	*p-*value
Hemodynamic instability score, median (IQR)	46 (25.5–91.5)	97 (75.5–118.5)	< 0.001
SBP > 160 mmHg			
Number of patients, n, (%)	34 (59.6%)	20 (45.5%)	0.156
Frequency, n, median (IQR)^**a**^	2.5 (1–5)	2 (1–2)	0.092
Duration (%), median (IQR)^**a**^	7.6 (2.3–12.6)	7.0 (2.3–15.2)	0.993
Maximum SBP (mmHg), median (IQR)	169 (147–194)	157 (127.5–183.3)	0.046
HR > 100 bpm			
Number of patients, n, (%)	18 (31.6%)	3 (6.8%)	0.002
Frequency, n, median (IQR)^**b**^	2 (1–5)	4 (3–4)	0.262
Duration (%), median (IQR)^**b**^	5.6 (1.8–18.4)	16.4 (4.0–16.4)	0.356
Maximum HR (bpm), mean (95%-CI)	90 (83.8–94.2)	80 (74.6–83.4)	0.012
MAP < 60 mmHg			
Number of patients, n, (%)	27 (47.4%)	27 (61.4%)	0.162
Frequency, n, median (IQR)^**c**^	2 (1–3)	2 (2–5)	0.025
Duration (%), median (IQR)^**c**^	4.8 (1.9–10.2)	7.1 (4.5–20)	0.013
Minimum MAP (mmHg), median (IQR)	60 (51.5–64.5)	56.5 (53–62)	0.41
HR < 50 bpm			
Number of patients, n, (%)	20 (35.1%)	20 (45.5%)	0.291
Frequency, n, median (IQR)^**d**^	1.5 (1–6)	3 (2–4.8)	0.142
Duration (%), median (IQR)^**d**^	9.9 (2.0–17.3)	16.9 (8.9–62.1)	0.038
Minimum HR (bpm), mean (95%-CI)	54 (51.4–56.6)	50 (47–53)	0.084

*TLA* Transperitoneal laparoscopic adrenalectomy, *PRA* posterior retroperitoneoscopic adrenalectomy, *IQR* interquartile range, *min* minute, *HR* heart rate, *MAP* mean arterial pressure, *SBP* systolic blood pressure, *bpm* beats per minute

a: in patients with a SBP of > 160 mmHg

b: in patients with a HR > 100 bpm

c: in patients with MAP of < 60 mmHg

d: in patients with a HR of < 50 bpm

**Fig. 2:**
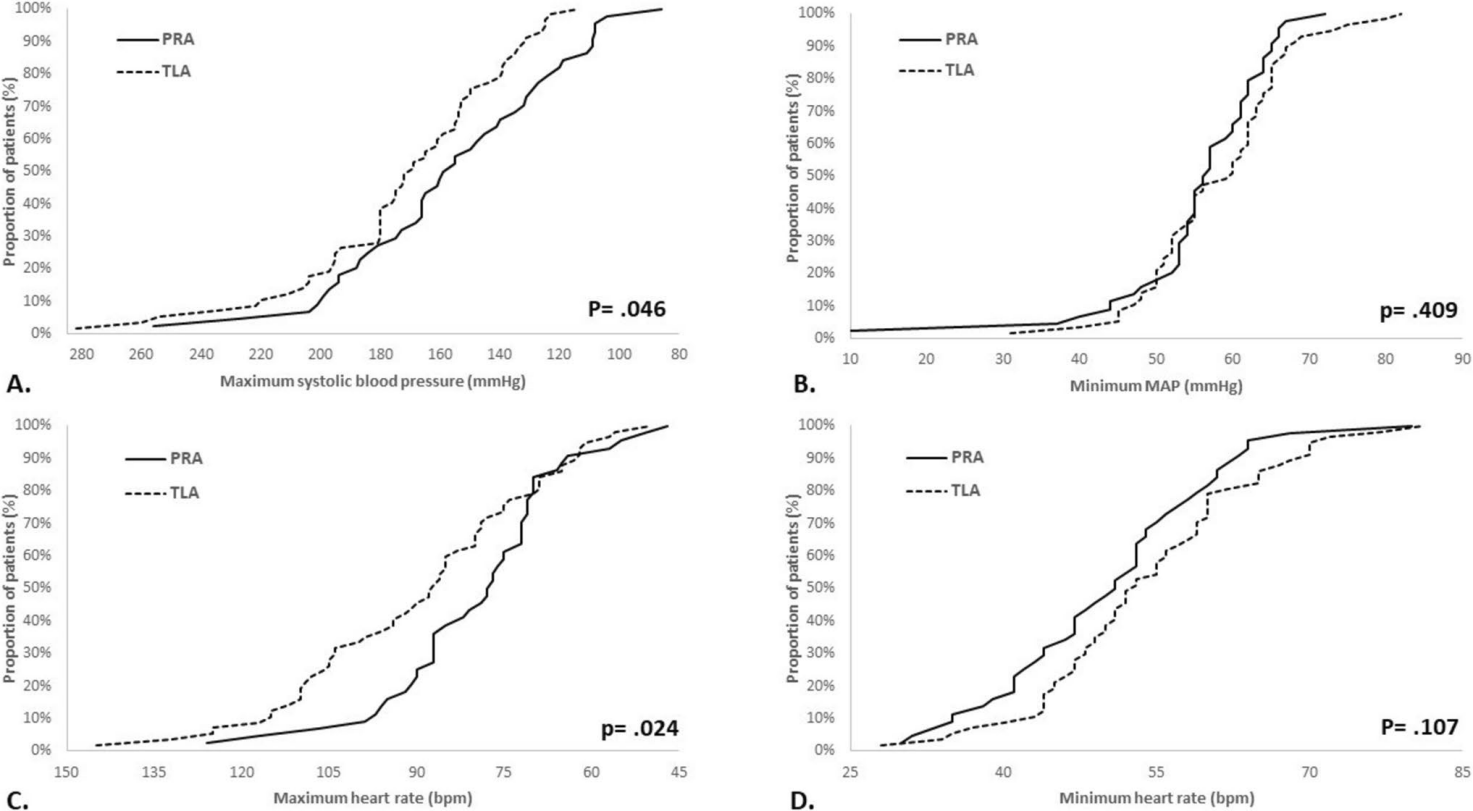
**A**–**D** Cumulative distribution of hemodynamic parameters in patients undergoing PRA and TLA.** A** Cumulative distribution of maximum blood pressure. **B** Cumulative distribution of minimum MAP. **C** Cumulative distribution of maximum HR. **D** Cumulative distribution of minimum HR

In the PRA group there were significantly more frequent and longer episodes of hypotension, and longer episodes of bradycardia. The frequency and duration of hypertensive episodes were not different between groups. However, the TLA group had significantly higher maximum SBP, more frequent episodes of tachycardia and higher maximum HR. Figure 2A–D shows the distribution of patients in each surgical group across the range of four major hemodynamic parameters.

To further investigate the potential effect of the different alpha-blockers, we performed a subgroup analysis specifically excluding patients receiving phenoxybenzamine. The hemodynamic instability score remained significantly higher in the PRA group than in the TLA group (101 (IQR: 86–119) vs 65 (35–98), *p* = 0.0002). To further investigate the effect of the learning curve of PRA on the HI-score, as it was introduced during the study period as a new technique, we calculated correlation coefficients for HI-score and year of surgery. We found no correlation between the year of surgery and HI-score (*ρ* = 0.22, *p* = 0.14).

### Drug and fluid administration

Table 6 presents the intraoperative drug and fluid administration for both groups. We found that PRA patients received more often and higher doses of vasoconstrictive drugs, particularly norepinephrine. Additionally, the fluid infusion rate was higher in the PRA group.

**Table 6:** Vasodilating and vasoconstrictive drugs administration in the surgical techniques

	Surgical technique		
	TLA N = 57	PRA N = 44	*p-*value
Vasodilating drug administration, n (%)	37 (64.9%)	17 (38.6%)	0.009
Number of different drugs administered,			*0.025*
1, n (%)	*27 (47.4%)*	*14 (31.8%)*	
2, n (%)	*10 (17.5%)*	*3 (6.8%)*	
MgSO_4_			
Use, n (%)	13 (22.8%)	4 (9.1%)	0.608
Dose (g), median (IQR)	3.2 (2.0–6.1)	4.225 (2.9–4.7)	0.296
Phentolamine			
Use, n (%)	35 (61.4%)	17 (38.6%)	0.023
Dose, g, (IQR)	7 (4–16)	4 (2–6)	0.018
Vasoconstrictive drug administration, n, (%)	45 (78.9%)	43 (97.7%)	0.017
Number of different vasoconstrictive drugs administered,			0.068
1, n (%)	39 (68.4%)	35 (79.5%)	
2, n (%)	5 (8.8%)	7 (15.9%)	
3, n (%)	1 (1.8%)	0	
Norepinephrine			
Use, n (%)	42 (73.7%)	41 (93.2%)	0.011
Dose (μg), median (IQR)	529 (294–827)	743 (411.5–1418)	0.032
Phenylephrine			
Use, n (%)	7 (12.3%)	5 (11.4%)	0.888
Dose (μg), median (IQR)	400 (200–1380	300 (165–450)	0.53
Epinephrin			
Use, n (%)	2 (3.5%)	2 (4.5%)	0.791
Dose (μg), median (IQR)	427 (20–427)	3.75 (2.5–3.75)	0.333
Ephedrine			
Use, n (%)	2 (3.5%)	2 (4.5%)	0.791
Dose (μg), median (IQR)	6.3 (5.0–6.3)	6.0 (2.0–6.0)	1.0
Dobutamine			
Use, n (%)	2 (3.5%)	0	0.209
Dose (μg), median (IQR)	6.3 (5.0–6.3)	0	
Atropine			
Use, n (%) Dose (μg), median	5 (8.8%) 0.5 (0.5–0.8)	4 (9.1%) 0.5 (0.5-.0.5)	0.956 0.73
Esmolol			
Use, n (%)	13 (22.8%)	6 (13.6%)	0.242
Dose (μg), median (IQR)	28 (16.7–60)	31 (10–109)	1.0
Fluid infusion rate (ml/h), mean (95%-CI)	798 (701–895)	1111 (993–1290)	0.002

*TLA* Transperitoneal laparoscopic adrenalectomy, *PRA* posterior retroperitoneoscopic adrenalectomy, *IQR* interquartile range

TLA patients on the other hand received more often and a higher number of vasodilating drugs, especially phentolamine.

## Discussion

We demonstrated that PRA is associated with a higher degree of hemodynamic instability than TLA in patients undergoing endoscopic adrenalectomy for PCC. Contrary to our initial hypothesis, we found more frequent and longer duration of hypotension in PRA, necessitating the use of vasoconstrictive medication and fluid infusion.

In several studies the association between hemodynamic instability and surgical technique in endoscopic adrenalectomy has been investigated [17–19]. Vorselaars et al. conducted a multinational study involving 341 patients from six centres in the United States, Europe, and Canada, to determine whether the operative technique affected intraoperative blood pressures during PCC surgery [19]. In their analysis they demonstrate that PRA has a sixfold higher risk for MAP < 60 mmHg compared to TLA. Their analysis also revealed that the medical centre was a significantly independent factor influencing blood pressure due to the lack of standardized preoperative treatment protocols. Nevertheless, the findings from Vorselaars et al. demonstrate a trend of lower MAP in PRA, which is consistent with our results. Ban et al. conducted a study comparing PRA with TLA in 53 patients with PCC and found lower intraoperative mean SBP (156 ± 27 vs 177 ± 23 mmHg, *p* = 0.0004) and diastolic blood pressures (88 ± 12 vs 102 ± 22 mmHg, *p* = 0.005) in patients undergoing PRA, which is also in agreement with our results [17]. Chen et al. reported a higher incidence of hypotension during PRA compared to TLA. In this study 108 patients were included who underwent adrenalectomy for various adrenal diseases [18]. Their analysis revealed that PRA was associated with a higher risk of a MAP < 60 mmHg, but this association was lost when PCCs were excluded. Additionally, it was found that PCC, along with PRA, was the strongest predictor of a MAP < 60 mmHg [18]. These findings suggest that the occurrence of hypotension during surgery could potentially be unique to PCC and related to preoperative alpha-adrenergic blockade. It is noteworthy that these studies employed various definitions of hemodynamic instability, which restricts the ability to make a comprehensive comparison with our results.

Differences in surgical positioning can potentially influence hemodynamics, because patients undergoing TLA are positioned in the lateral decubitus position, while those undergoing PRA are placed in the jack-knife position. Borodiciene et al. compared the hemodynamic changes during anorectal surgery in lithotomy and jack-knife position in 104 adult patients and measured the cardiac output, cardiac index, and systemic vascular resistance using impedance cardiography. They demonstrate that the jack-knife position, after low-dose spinal anaesthesia, produced a statistically significant reduction of cardiac output and cardiac index, with an increase in systemic vascular resistance compared to insignificant changes in the lithotomy position [20]. These findings contradict with the results of the study conducted by Chen et al., which did not observe a decrease in MAP in PRA when adjusted for pheochromocytoma [18]. This discrepancy could be attributed to the heterogeneity of the patient population in Chen’s study or to differences in the surgical and anaesthesiologic techniques employed in Borodiciene ‘s study. Nevertheless, the findings of the study by Borodiciene et al. highlight an intriguing and potentially influential factor contributing to the hypotension we observed in patients with PRA.

Considering the working space, along with its associated gas pressure column differences, as a possible determinant of the hemodynamic changes, Myre et al. revealed that during pneumoperitoneum, both venous and arterial norepinephrine levels were considerably higher than before pneumoperitoneum [21]. The authors hypothesize that this increase in norepinephrine could be caused by local activation of the sympathetic nervous system in the abdominal region [21]. While this study did not perform a comparison to pneumoretroperitoneum, it is plausible that the sympathetic nerve stimulation associated with TLA could potentially explain our observed increases in SBP and HR, as well as the higher need of vasodilating drug administration in the TLA group.

Our study has several strengths. It was conducted as a single-centre study with a large cohort of patients, utilizing uniform protocols for perioperative care. Also, the use of the HI-score, a validated scoring system, provides a comprehensive overview of the patient’s hemodynamic condition, enabling accurate comparison of outcomes between the different groups. The minute-by-minute registration of intraoperative data enabled us to collect precise and detailed information on hemodynamic instability during the procedure. Finally, the use of an automated HI-score calculator minimized the risk of human error in the calculation process and ensured that our results are reliable and robust.

There are several limiting factors in our study related to its retrospective nature. Our study was limited to adrenal PCC cases, therefore, our results cannot be extrapolated to extra-adrenal paraganglioma. Additionally, our eligibility criteria for PRA suggest that these patients had relatively small tumours and little obesity-related comorbidity, which could have resulted in selection bias. The baseline characteristics including tumour size, however, were not different between the groups. Patients were not randomized between PRA and TLA, but the surgical approach was determined by eligibility and the preference of the surgeon. Furthermore, the TLA group had more cardiovascular events prior to surgery than PRA. It is unclear how this may have affected hemodynamic differences. In any case, the preoperative medical management was similar between the groups. Furthermore, PRA became the preferred technique from 2011 onwards, which could potentially introduce historical bias. However, our analysis showed no association between the date of surgery and the HI-score, suggesting that surgical experience did not significantly impact intraoperative hemodynamics.

Another potential source of historical bias is the transition in preoperative preparation from phenoxybenzamine to doxazosin. Our findings indicated a higher frequency of hypotensive episodes in the PRA group, where a larger proportion of patients were administered doxazosin compared to the TLA group (91% vs. 67%). Interestingly, doxazosin is generally associated with less hypotension compared to phenoxybenzamine. Therefore, our results in PRA suggest the surgical approach to be of influence.

Overall, our study demonstrated the hemodynamic differences between surgical techniques in patients undergoing endoscopic surgery for PCC, highlighting more hypotension, fluid infusion and vasoconstrictive medication use in patients undergoing PRA compared to TLA. However, the existing guidelines for preoperative management of PCC were developed during the time that TLA was the most commonly applied surgical technique [14]. Given our findings, we urge for a more patient-tailored preoperative alpha-adrenergic blockade strategy that considers both individual patient factors and anticipated surgical technique. Prospective clinical trials are necessary to optimize these preoperative alpha-blockade strategies, taking cardiovascular comorbidity into account and to demonstrate their effect on intraoperative hemodynamic instability.

## Supplementary Information

Below is the link to the electronic supplementary material.

Supplementary file1 (DOCX 22 KB)

## Data Availability

Data will be made available upon written request.

## Abbreviations

PCC: Pheochromocytoma
TLA: Transperitoneal laparoscopic adrenalectomy
PRA: Posterior retroperitoneoscopic adrenalectomy
HI-score: Hemodynamic instability score
SBP: Systolic blood pressure
MAP: Mean arterial pressure
bpm: Beats per minute
HR: Heart rate
