# Supplementary material for: Impact of surgical technique on hemodynamic instability in patients with pheochromocytoma: a single-centre retrospective cohort study
Source: Surg Endosc. 2025 May 15;39(7):4166–76. doi: 10.1007/s00464-025-11794-2 (PMC12222387; doi:10.1007/s00464-025-11794-2)
Supplement: Supplementary file 1 — Supplementary file1 (DOCX 22 KB) [file 464_2025_11794_MOESM1_ESM.docx]

**DATA SUPPLEMENT**

**This appendix has been provided by the authors to give readers additional information about their work.**

**Supplement to: Impact of Surgical Technique on Hemodynamic Instability in Patients with Pheochromocytoma: A Single-Centre Retrospective Cohort Study**Authors: Amir-Hossein Chaman Baz, Julie van de Wal, Simone A.A. Willems, Frank d’Ancona, Xiaoye Zhu, Henri J.L.M. Timmers, Johan F. Langenhuijsen

**Anaesthetic management**

In preparation for the surgical procedure, preoperative anxiolytic medication was administered to the patient both on the evening before and in the morning of surgery. Additionally, a dose of 1000 mg paracetamol was given orally. The last dose of alpha-blocker was administered at the preoperative night or in the morning of surgery depending on starting time of the operation.

Before surgery, peripheral intravenous and arterial lines were inserted under local anaesthesia. Preoxygenation was carried out, followed by induction of anaesthesia. Muscle relaxation was achieved, and intubation was performed while ensuring that the patient reached an adequate depth of anaesthesia to prevent a hypertensive crisis. The maintenance of anaesthesia was achieved using propofol or inhalational agents. The aim was to maintain a train-of-four (TOF) ratio below 2. During surgery, intraoperative hemodynamic parameters and drug and fluid administration were registered minute-by-minute.

Hypertension:

Several medications were used to correct for hypertension (systolic blood pressure (SBP) > 160 mmHg). Alpha-adrenergic antagonists such as phentolamine were administered intravenously in doses of 1 to 2 mg. Magnesium sulphate was occasionally used, for vasodilation and inhibition of catecholamine release from the adrenal glands. A loading dose of 50 mg/kg was given, followed by a continuous infusion of 2 g/hour. Calcium antagonists were used for arterial vasodilation and inhibition of catecholamine release, with a recommended dosage of 1-3 μg/kg/min. Venous vasodilators like nitroprusside (0.5-5 μg/kg/min) or nitro-glycerine (0.5-10 μg/kg/min) were employed in some cases.

Tachycardia:

In the case of tachycardia (heart rate (HR) > 100/min), a selective beta-blocker with a short duration of action like esmolol, was used. Loading doses of 10-20 mg (500 μg/kg in 1 minute) were administered, followed by an infusion of 50-300 μg/kg/minute. In cases where there was an epinephrine (dopamine)-secreting tumour, a dose of 20 mg IV was given just prior to surgical manipulation.

Arrythmias:

In the event of arrhythmias during surgery, several medications were employed. Magnesium sulphate was a preferred option due to its ability to inhibit catecholamine release from the adrenal glands and its antiarrhythmic properties. A loading dose of 50 mg/kg was administered, followed by a continuous infusion of 2 grams per hour. Amiodarone was another choice, involving a loading dose of 5 mg/kg over 30 minutes, followed by a maintenance dose of 15 μg/kg/minute. Additionally, lidocaine was used, for its short duration of action without negative inotropic effects. A dose of 1 mg/kg was typically administered.

Hypotension:

In the case of hypotension (mean arterial pressure (MAP) < 60 mmHg), supplemental volume was provided using Ringer's lactate solution. Medications such as norepinephrine (at a dose of 0.05-0.1 μg/min/kg) or phenylephrine (titrated with doses of 100 μg) were used.

*Supplementary table 1.* *Distribution of minimally invasive adrenalectomies performed by each urologist from 2007-2022 for various adrenal diseases.*

|  | Surgical technique | |  |
| --- | --- | --- | --- |
|  | TLA *n= 496* | PRA *n= 429* | Total n= 925 |
| Urologist A | 278 (42%) | 378 (58%) | 656 |
| Urologist B | 171 (100%) | 0 | 171 |
| Urologist C | 47 (48%) | 51 (52%) | 98 |

TLA = transperitoneal laparoscopic adrenalectomy; PRA = posterior retroperitoneoscopic adrenalectomy. All data is presented as n, (% total adrenalectomies for that urologist).
